# Near fatal intoxication by nicotine and propylene glycol injection: a case report of an e-liquid poisoning

**DOI:** 10.1186/s40360-019-0296-8

**Published:** 2019-05-10

**Authors:** Mhedi Belkoniene, Jennifer Socquet, Denise Njemba-Freiburghaus, Cyril Pellaton

**Affiliations:** 1 Department of Internal Medicine, Hôpital Neuchâtelois, Neuchâtel, Switzerland; 2 Intensive Care Unit, Hôpital Neuchâtelois, Neuchâtel, Switzerland

**Keywords:** E-liquid, Nicotine, Propylene glycol, Intoxication, Injection

## Abstract

**Background:**

Concern about intoxication by e-liquid is growing as calls to poison control centers have increased since their introduction. Only three cases of intoxication by injection have been reported worldwide. Our case is unique because of the precise follow-up of a patient who survived a lethal dose of self-injected e-liquid, without other co-intoxication.

**Case presentation:**

A 51-year-old male presented to the Emergency Department after injecting himself intravenously (IV) in the forearm with 10 mL of e-liquid (1000 mg of nicotine diluted in propylene glycol). An agitation phase was followed by coma and bradypnoea requiring mechanical ventilation. The patient developed a transitory neurological impairment with the appearance of tetraparesis, gaze palsy and myoclonus due to nicotinic syndrome. The arterial blood gas (ABG) analysis confirmed uncompensated lactic acidosis with an elevated anion gap, which is an expected effect of propylene glycol. The toxicology screen indicated the presence of nicotine and cotinine in the blood and excluded the presence of concomitant intoxication. The patient recovered without sequelae.

**Conclusion:**

Even a small quantity of intravenous (IV) e-liquid can lead to an acute intoxication and fatal outcomes due to the toxic effects of nicotine. This case might help emergency doctors cope with acute intoxication by injection of e-liquid and increase their comprehension of the two main substances, nicotine and propylene glycol with overview of their pharmacodynamics and kinetic effects.

## Background

The popularity of electronic cigarettes (e-cigarette) has increased since its commercialisation in 2004 [1]. Despite being considered safer than conventional nicotine delivery systems [2, 3], the number of reported intoxications has significantly increased worldwide [4, 5]. In Europe, The European Tobacco Product Directive (TPD) reported 277 incidents in 10 countries between 2012 to 2015 [6] while 1414 cases were registered in 2013 in the United States [1] alone. While the majority of cases are not serious, 6,3% have more severe outcomes [6], sometimes fatal [1, 3].

No studies about the safety profile of e-liquid, especially in the case of acute intoxication, were conducted before releasing it on the market for use [7]. Today’s knowledge only comes from separate studies of the toxicity of the two active components, nicotine and propylene glycol (PG) [2, 7–10], as well as from a few case reports and national reports of adverse events [1, 6]. The toxicity of PG has been demonstrated in in vitro tests [11] and some case reports about suspected intoxication by PG as a diluent for IV drugs like lorazepam [11, 12]. The toxicity of nicotine has been widely studied when inhaled, ingested and in transdermal use, but never when injected [7, 13]. The population concerned by e-liquid intoxication consists of paediatric patients (close to 35% of reported events) principally unintentional ingestion [1, 3, 5, 6] and adults with psychiatric illnesses using e-liquid in suicide attempts [8, 14–18]. E-liquid injection has been documented in only three cases. In 2010, Hagiya [19] described the first case of nicotine injection leading to mild toxicity: nausea, palpitations, vomiting and abdominal pain. In 2014, Thornton [8] reported the case of a man found dead with a suicide note indicating self-injection of e-liquid [4]. In 2014, Sommerfeld [9] described a case of e-liquid injection with concomitant ethanol intoxication with resultant transient coma and bradypnoea.

In this report, we present the clinical case of a near fatal e-liquid injection, with no other concomitant intoxication, and precise monitoring. This case might help physicians diagnose and manage this new type of intoxication by understanding the mechanisms of nicotine and PG in case of injection.

## Case presentation

A fifty-one-year-old man known for active e-cigarette smoking and history of cigarette smoking, type 2 diabetes mellitus and a personality disorder was brought to the Emergency Department 30 min after injecting himself intravenously in his right forearm with 10 ml of e-liquid with 100 mg/ml of nicotine diluted in propylene-glycol in a suicidal attempt.

On arrival, the patient already complained of diffuse abdominal cramps. He confirmed the intravenous injection of 10 ml of e-liquid in the forearm and brought the product with him. Initial vital signs showed a heart rate of 139 beats per minute, a blood pressure of 170/113 mmHg, a temperature of 36 °C (96.8 °F), a respiratory rate of 41 breaths per minute and a saturation of 100% on room air. Physical examination was irrelevant except for psychomotor agitation and mydriatic pupils poorly responsive to light. No local reaction was visualised around the injection site (on the forearm). The ABG showed a mixed acidobasic disorder with metabolic acidosis and respiratory alkalosis (pH 7.56, pCO2 1.31 kPa, pO2 15,8 kPa and bicarbonate 8.9 mmol/l, lactate 11.1 mmol/l). The anion gap was elevated (31.1 mmol/l) as was the osmolar gap, reaching 16 mOsm/kg. Venous blood analysis showed hypokalaemia (3 mmol/l) and hypophosphataemia (0.23 mmol/l). The ECG showed a sinusal tachycardia without repolarisation changes and the troponins were negative. The patient was initially rehydrated; IV potassium and phosphate infusion was initiated and morphine was administrated to control pain. Two hours post-injection the patient became stuporous with bradypnoea and desaturation. The subsequent ABG showed persistent uncompensated lactic acidosis with the appearance of alveolar hypoventilation, (pH 7.22, pCO2 5.25 kPa, bicarbonate 16.6 mmol/l and lactate 5.7 mmol/l). The patient fell into a coma and was quickly transferred to the Intensive Care Unit (ICU) where he was immediately intubated using rapid sequence induction (etomidate, succinylcholine and fentanyl). For the next 3 h, the patient was not sedated but remained in a profound coma, being unarousable (GCS 3/15) and showing insufficient spontaneous respiration needing controlled ventilation. He presented periodic myoclonic movements of both lower limbs with no abnormal movement of the upper body without improvement after 1 mg of IV clonazepam. Seven hours post-injection the patient recovered spontaneous ventilation and woke up progressively. Ten hours post-injection, the patient was alert and answered simple questions by shaking his head, but his pupils were still mydriatic and poorly responsive to light. We noted a right lateral gaze palsy and flaccid tetraparesia 2/5 with hypoactive deep tendon reflexes, thus preventing extubation. A brain CT excluded any cerebral lesion. Eleven hours post-injection the patient showed complete recovery of motor response and normalisation of deep tendon reflexes allowing extubation.

His mean arterial blood pressure stayed in the normal range without vasopressive agents. We observed a sinus tachycardia (110–130 bpm) and short runs of atrial tachycardia. No ventricular arrhythmias were noted during monitoring. The troponins peaked at 1450 ng/l 24 h post-injection. We concluded a type II myocardial infarction due to sympathetic overstimulation. Daily aspirin was introduced and an ambulatory cardiac assessment was organised.

We noted polyuria up to 400 ml/h with a normal urine analysis and a urine osmolality of 620 mOsm/kg. IV hydration was initiated to compensate for the polyuria, which resolved spontaneously after 8 h. Metabolic acidosis with hyperlactataemia persisted on serial ABGs but it normalised after 11 h.

**Fig. 1:**
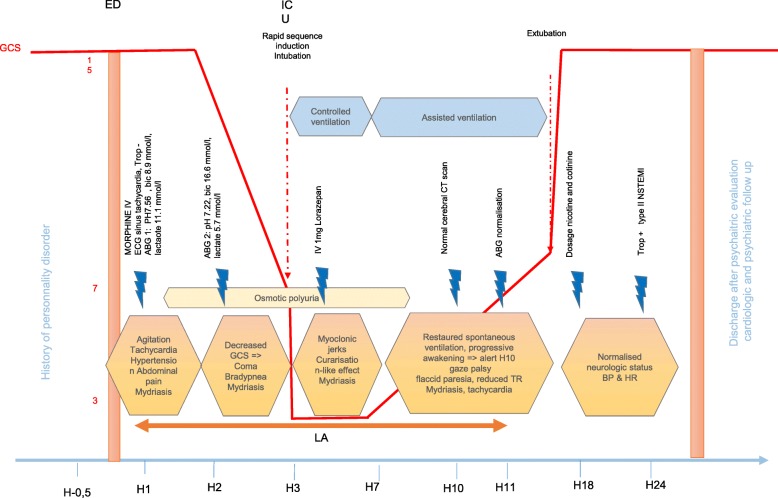
Timeline. Legend: ABG: arterial blood gas, Bic: Bicarbonate, BP: Blood pressure, ECG: electrocardiography, HR: Heart rate, TR: tendinous reflex, Trop: troponin, CT scan: computed tomography

Cardiologic and neurologic monitoring was performed for 24 h. The patient was discharged after a psychiatric evaluation and the organisation of an out-patient follow-up. A summarized view of the patient’s evolution is shown in Fig. 1.

Knowing the concentration of nicotine and the quantity injected of a labelled product, we did not ask for more investigations. We confirmed the poising by performing nicotine and cotinine dosages, which were 18 h post-injection12 mcg/l and 3210 mcg/l respectively. The toxicology screen for other common drugs was negative. The presence of Propylene glycol was confirmed as an adjuvant on the label of the bottle the patient brought with him, but its dosage was not available in several universitary laboratories.

## Discussion

Few cases of acute intoxication with known quantity of nicotine or precise plasmatic concentration have been published (Table 1). As shown, it is very difficult to create a common profile of toxicity of e-liquid because of the heterogenic nature of cases described: different route of administration, concomitant intoxication, time to sampling. Unlike other poisoning routes, e-liquid acts differently when injected, as the two main molecules, nicotine and PG, reach an hyperacute plasmatic peak, which is well described in our case, as there was no concomitant intoxication.

**Table 1:** Overview of Nicotine intoxication cases and their toxicological results

CASE	Route IV^a^ or PO^b^	Co intoxication	Amout of *-Nicotine use (mg)	Time to sample (hours)	Plasmatic nicotine level μg/l	Plasmatic cotinine level μg/l	Clinical findings	Issue
Thornton [8]	IV	Amphetamine	400	x	2000	2100	Cardiac asystoly	†
Sommerfeld Case 1 [9]	Po	none	372	1.5	96	2800	Nausea, vomiting, Hypotension, bradycardia	survived
Sommerfeld Case 2 [9]	IV	Ethanol (70 mg/ml)	128.8	2	800	2400	Coma, bradypnea, nausea	survived
Martin kleish [21]	Po	none	2288.5	2	528	176	Tachycardia, hypertension, myosis, abominal pain	survived
Solarino [10]	Po	Ethanol 2,1 g/l	1000 (in stomach)	x	2200	2200	No data	†
Our case	IV	none	1000	18.5	12	3200	tachycardia, coma, bradypnea,mydriasis, myoclonus, flaccid paresia, type 2 NSTEMI	survived

^a^IV: injection

^b^Po: ingestion

† Death

X time not known

## Nicotine

Nicotine is an amine present in tobacco and its derivates such as chewing gum, patches and the e-liquid of e-cigarettes [20]. The bioavailability after inhalation or injection is near complete and leads to a rapid peak concentration in the blood. When ingested, the bioavailability only reaches 20% because of limited absorption, the vomiting reflex and a first enteric passage. Nicotine is quickly metabolised, up to 75%, by the liver cytochrome CYP2A6 into cotinine and to a minor extent by glucuronidation [1, 9]. The remaining nicotine is filtered by the kidney. Cotinine, an inactive metabolite, has a longer half-life (15-19 h) compared to nicotine (2-3 h), which makes it a perfect marker to confirm nicotine toxicity even hours after exposure [9, 10, 20]. The volume of distribution is between 2 and 3 l/kg with high brain affinity [8].

**Fig. 2:**
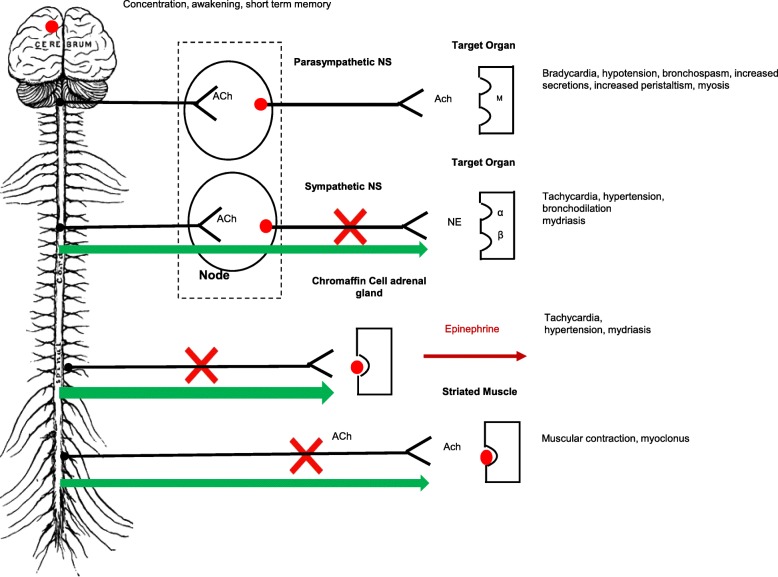
Cholinergic Syndrome. Legend: Ach: Acetylcholine, NE: Norepinephrine, M: muscarinic receptor, : Activation with overstimulation (early phase), : Inhibition with desensitization (late phase), : Nicotinic receptor

Nicotine mimics the effect of acetylcholine by stimulating the nicotinic cholinergic receptors (nAChRs). The various effects of nicotine are due to the ubiquity of these receptors in the central nervous system (CNS), the autonomic nervous system (ANS) and the neuromuscular junction (NMJ) [20] (Fig. 2).

In the CNS, the stimulation of nAChRs releases a variety of neurotransmitters: dopamine, norepinephrine, acetylcholine, glutamate, endorphins and GABA (associated with concentration, awakening, short term memory) [20].

In the ANS, nicotine stimulates nAChRs in both the sympathetic and parasympathetic systems [5, 20]. The stimulation of the parasympathetic system induces nausea, vomiting, increased salivation, bradycardia, hypotension, respiratory bronchospasm and myosis [8–10, 21]. In the sympathetic system, it promotes the release of epinephrine by the adrenal medulla causing resultant tachycardia and hypertension [21]. Nicotine thereby induces a mixed response with both sympathetic and parasympathetic effects [10, 22]. On the NMJ, nicotine induces muscle contraction.

The relation between the dose of nicotine and its effects is complex [10]. Low doses of nicotine stimulates the nAChRs, while higher doses, as in the case of injection, induce a biphasic clinical reaction [1, 23]. In the early phase, stimulation of the nAChRs occurs in both systems of the ANS like we observed in our patient presenting subsequent hypertension, tachycardia, abdominal pain, tremor and agitation. In the late phase (2 h post-injection in our case), a global desensitisation of all nAChRs appears, causing central nervous system depression, ganglionic blockade and curare-like effects on the NMJ [9, 10] with coma and respiratory failure [21, 23].

The suspected lethal dose of nicotine is 30–60 mg, which corresponds to an oral lethal dose (LD50) of approximately 0.8–1 mg/kg [8, 9, 22] (less than 1 ml of the product used by our patient) [22]. This range is considered controversial [22], with a possible new LD50 value ranging from 500 to 1000 mg. The analysis of fatal cases suggests a possible lethal plasmatic nicotine level of 4 mg/l [22].

Without a specific antidote available, the appropriate treatment of a nicotinic intoxication is cardio-pulmonary support [3, 21] and ICU monitoring because of the high risk of coma. Atropine could conteract bradycardia [21, 23]. In case of ingestion, activated charcoal and induction of emesis could prevent further absorption [3, 21]. As nicotine is excreted by the kidneys, a good urine output is necessary [23], hemodialysis is not recommended [3].

## Propylene glycol

Propylene glycol (PG) is used as a solvant in numerous products such as food, cosmetics and pharmaceuticals and is considered safe, eventhough PG is associated in several cases with severe toxicity when added as a solvent to IV drugs such as certain benzodiazepines used in the ICU [12]. PG is rapidly absorbed by the intestinal tract with a maximal plasmatic peak [12] 1 h after ingestion. Its plasmatic half-life is 1.4 to 3.3 h. Approximately 55% of PG undergoes oxidation to propionaldehyde and pyruvic, acetic and lactic acid in the liver, while the remainder is excreted unchanged in the urine [8]. In the case of PG intoxication, lactic metabolic acidosis with an elevated anion gap is observed. Another potential effect is an increased blood osmolality sometimes associated with a severe osmolar gap. The clinical manifestations of PG intoxication are various and multisystemic; it can induce CNS depression with coma and sometimes seizures because of an accumulation of D-lactate in the brain [24] and cardiac arrhythmias [25]. Renal impairment has also been documented but the cause remains unclear [26]. Some studies have demonstrated that PG injures proximal tubular cells, leading to impaired renal acidification. The toxic dose for an adult with normal renal and hepatic function is 1 g/l [26]. According to Arroglia et al. the osmolar gap could be used as a surrogate to estimate the PG concentration [25]. According to Kraut et al., PG intoxication does not warrant specific treatment because of its rapid metabolism and they propose bicarbonate infusion and hydration [26]. Other authors have reported severe intoxications that were resistant to the above mentioned therapy and where infusion of fomepizol to stop metabolite production or haemodialysis could be considered [12, 24, 25].

Our patient presented a metabolic acidosis and an osmotic diuresis up to 400 ml/h, probably caused by the PG intoxication. In our case, the plasmatic concentration of PG was not available, but we observed signs of intoxication with moderate to severe hyperlactataemic acidosis and osmotic diuresis.

With the growth of e-liquid use, physicians will be confronted with more cases of life-threatening intoxications by nicotine and PG. Cases of e-cigarette injection are still rare but reveal the unique effects of nicotine and PG by the IV route. The lethal dose in the case of injection is not known and, in most hospitals, no quick or reliable tests are available to confirm such intoxication. Nicotine and PG are not part of the common toxicology screen set and has to be specifically requested. Non-specific symptoms could delay diagnosis and therefore the sampling. Our case is limited by the fact that the nicotine level was measured 18 h after the injection, thus it is difficult to establish a correlation between the clinical effects and the plasmatic dose, but the typical biphasic clinical presentation and the high blood cotinine level 18 h post injection indicate a high concentration of nicotine in the blood in the first hours. Knowing the typical biphasic pattern in case of nicotine injection can help to predict the expected CNS depression and respiratory failure due to respiratory muscle paresis approximately 3 h after injection. This case report presents the natural course of an acute nicotine and PG intoxication, its effects, a clear timeline and the treatment provided. E-liquids can be lethal when misused. More systematic studies would be helpful to understand the danger of this new substance and the regulations it should be submitted to.

## Abbreviations

ABG: Arterial blood gas
ANS: Autonomic nervous system
CNS: Central nervous system
ICU: Intensive care unit
IV: Intravenous
LD50: Oral lethal dose
nAchRs: Nicotinic cholinergic receptors
NMJ: Neuromuscular junction
PG: Propylene glycol
